# Recent Progress on Nonlinear Schrödinger Systems with Quadratic Interactions

**DOI:** 10.1155/2014/214821

**Published:** 2014-03-31

**Authors:** Chunhua Li, Nakao Hayashi

**Affiliations:** ^1^Department of Mathematics, College of Science, Yanbian University, No.977 Gongyuan Road, Yanji, Jilin 133002, China; ^2^Department of Mathematics, Graduate School of Science, Osaka University, Toyonaka, Osaka 560-0043, Japan

## Abstract

The study of nonlinear Schrödinger systems with quadratic interactions has attracted much attention in the recent years. In this paper, we summarize time decay estimates of small solutions to the systems under the mass resonance condition in 2-dimensional space. We show the existence of wave operators and modified wave operators of the systems under some mass conditions in *n*-dimensional space, where *n* ≥ 2. The existence of scattering operators and finite time blow-up of the solutions for the systems in higher space dimensions is also shown.

## 1. Introduction

In this paper we survey recent progress on asymptotic behavior of solutions to nonlinear Schrödinger system,

(1) i∂tvj+12mjΔvj=Fj(v1,…,vl)+Gj(vj),t∈ℝ,  x∈ℝn,vj(0,x)=ϕj, x∈ℝn,

based on papers [1–9], where 1 ≤ *j* ≤ *l*, $\bar{v_{j}}$ is the complex conjugate of *v*_*j*_, *m*_*j*_ is a mass of particle, and nonlinearities have the form

(2) $$\begin{array}{c} F_{j}\left(v_{1},\ldots ,v_{l}\right)=\underset{1\leq m\leq k\leq 2l}{\sum }\lambda_{m,k}^{j}v_{m}v_{k},\\ G_{j}\left(v_{j}\right)=\mu_{j}\left|v_{j}\right|v_{j} \end{array}$$

with

(3) vm,vk,vj∈{v1,…,vl,v1¯,…,vl¯}={v1,…,vl,vl+1,…,v2l}, λm,kj,  μj∈ℂ.

Nonlinear Schrödinger systems with quadratic interactions are physically important subjects (see, e.g., [10] and references cited therein). The quadratic nonlinearities of nonlinear Schrödinger systems in two space dimensions are interesting mathematical problems since they are regarded as the borderline between short range and long range interactions. In this case, asymptotic behavior of solutions to nonlinear systems is different from that to linear systems under mass resonance conditions and is the same as that to linear systems under mass nonresonance conditions. Namely, it is impossible to find solutions of nonlinear systems in the neighborhood of those of linear systems under mass resonance conditions.

If *F*_*j*_(*v*_*j*_) ≡ 0, then we have a single nonlinear Schrödinger equation:

(4) $$\begin{array}{c} i\partial_{t}v_{j}+\frac{1}{2m_{j}}\Delta v_{j}=\mu_{j}\left|v_{j}\right|v_{j}. \end{array}$$

There are a lot of works on this subject since the work by Ginibre and Velo [11] which is considered a milestone of the field. We refer the text book by Cazenave [12] concerning the development on studies of (4) for details.

It is interesting to compare (1) with the system of nonlinear Klein-Gordon equations

(5) 12c2mj∂t2uj−12mjΔuj+mjc22uj =−Fj(u1,…,ul)−Gj(uj)

in (*t*, *x*) ∈ ℝ × ℝ^*n*^ for 1 ≤ *j* ≤ *l*, under the gauge invariant condition

(6) $$\begin{array}{c} F_{j}\left(v_{1},\ldots ,v_{l}\right)=e^{im_{j}\theta }F_{j}\left(e^{-im_{1}\theta }v_{1},\ldots ,e^{-im_{l}\theta }v_{l}\right) \end{array}$$

for any *θ* ∈ ℝ, where *c* is the speed of light. If we let *u*_*j*_ = *e* ^−*itm*_*j*_*c*^2^^ *v*_*j*_ in (5), then by the condition (6) we find that *v*_*j*_ satisfies

(7) 12c2mj∂t2vj−i∂tvj−12mjΔvj  =−eimjθFj(e−im1θv1,…,e−imlθvl)   −eimjθGj(e−imjθvj)  =−Fj(v1,…,vl)−Gj(vj)

with *θ* = *tc* ^2^ for 1 ≤ *j* ≤ *l*. Therefore nonrelativistic version of (5) can be obtained by letting *c* → *∞* in (7) formally, which is (1). The first breakthrough on asymptotic behavior of solutions to (5) with *G*_*j*_ ≡ 0 was made by Klainerman [13] and Shatah [14] independently when *n* = 3. Their result was improved by a paper [15].

It is natural to require the **L** ^2^(ℝ^*n*^) conservation law of solutions to (1) from the point of view of quantum mechanics. A sufficient condition is

(8) Im⁡∑j=1lcjFjvj¯=0,

where *c*_*j*_ > 0 for 1 ≤ *j* ≤ *l*; then we have **L** ^2^(ℝ^*n*^) conservation law and as a result global existence in time of solutions to (1) is obtained by combining the conserved identity and the Strichartz estimate for *n* ≤ 4 (see [16] in which a single equation was considered and the proof used in [16] works for the system).

We now introduce some function spaces to present exact statements of our results. For any *m*, *s* ∈ ℝ and 1 ≤ *p* ≤ *∞*, weighted Sobolev space **H**_*p*_^*m*,*s*^(ℝ^*n*^) is defined by

(9) $$\begin{array}{c} H_{p}^{m,s}\left({\mathbb{R}}^{n}\right)=\left\{f\in {𝕊}^{\prime }\left({\mathbb{R}}^{n}\right);{||f||}_{H_{p}^{m,s}({\mathbb{R}}^{n})}<\infty \right\}, \end{array}$$

where

(10) $$\begin{array}{c} {||f||}_{H_{p}^{m,s}({\mathbb{R}}^{n})}={||{\left(1-\Delta \right)}^{m/2}{\left(1+x{|}^{2}\right)}^{s/2}f||}_{L^{p}({\mathbb{R}}^{n})}. \end{array}$$

We write **H**_2_^*m*,*s*^(ℝ^*n*^) = **H** ^*m*,*s*^(ℝ^*n*^) and **H** ^*m*,0^(ℝ^*n*^) = **H** ^*m*^(ℝ^*n*^) for simplicity. **L** ^*p*^(ℝ^*n*^) denotes the usual Lebesgue space with the norm

(11) $$\begin{array}{c} {||\phi ||}_{L^{p}({\mathbb{R}}^{n})}={\left(\int_{{\mathbb{R}}^{n}}{\left|\phi \left(x\right)\right|}^{p}dx\right)}^{1/p} \end{array}$$

if 1 ≤ *p* < *∞* and

(12) $$\begin{array}{c} {||\phi ||}_{L^{\infty }({\mathbb{R}}^{n})}=\text{ess}\cdot \underset{x\in {\mathbb{R}}^{n}}{\sup}\left|\phi \left(x\right)\right|. \end{array}$$

By ${\dot{B}}_{p,q}^{s}\left({\mathbb{R}}^{n}\right)$ we denote the homogeneous Besov space with the seminorm

(13) ||ϕ||B˙p,qs(ℝn) =(∫0∞λ−1−σqsup⁡|y|≤λ ∑|α|=[s]||∂α(ϕy−ϕ)||Lp(ℝn)qdλ)1/q,

where *s* = [*s*] + *σ*, 0 < *σ* < 1, *ϕ*_*y*_(*x*) = *ϕ*(*x* + *y*),  1 ≤ *p*, *q* ≤ *∞*, and [*s*] is the largest integer less than *s*. It is known that ${\dot{B}}_{2,2}^{s}\left({\mathbb{R}}^{n}\right)={\dot{H}}^{s,0}\left({\mathbb{R}}^{n}\right)$ (see [17]). We let **C**(**I**; **E**) be the space of continuous functions from an interval **I** to a Banach space **E**. Different positive constants might be denoted by the same letter *C*. The homogeneous Sobolev spaces ${\dot{H}}^{m,0}\left({\mathbb{R}}^{n}\right)$ and ${\dot{H}}^{0,s}\left({\mathbb{R}}^{n}\right)$ are defined by

(14) H˙m,0(ℝn) ={f∈𝕊′(ℝn);||f||H˙m,0(ℝn)=||(−Δ)m/2f||L2(ℝn)<∞},H˙0,s(ℝn) ={f∈𝕊′(ℝn);||f||H˙0,s(ℝn)=|||x|sf||L2(ℝn)<∞},

respectively. We define the dilation operator by

(15) $$\begin{array}{c} \left(D_{\delta }\phi \right)\left(x\right)=\frac{1}{{\left(i\delta \right)}^{n/2}}\phi \left(\frac{x}{\delta }\right) \end{array}$$

for *x* ∈ ℝ^*n*^ and *δ* ≠ 0 and define *E* = *e* ^−(*i*/2)*t*|*ξ*|^2^^ and *M* = *e* ^−(*i*/2*t*)|*x*|^2^^ for *t* ≠ 0.

Evolution operator *U*_*δ*_(*t*) is written as

(16) $$\begin{array}{c} \left(U_{\delta }\left(t\right)\phi \right)\left(x\right)=M^{-1/\delta }D_{\delta t}\left(ℱM^{-1/\delta }\phi \right)\left(x\right), \end{array}$$

where *ℱ*, *ℱ* ^−1^ are the Fourier transform and the inverse Fourier transform, respectively. We also have

(17) $$\begin{array}{c} U_{\delta }\left(-t\right)\phi \left(x\right)=i^{n}M^{1/\delta }\left({ℱ}^{-1}E^{\delta }D_{1/\delta t}\phi \right)\left(x\right). \end{array}$$

The operator *J*_1/*m*_*j*_,*k*_ = *x*_*k*_ + *i*(*t*/*m*_*j*_)∂_*k*_ = *U*_1/*m*_*j*__(*t*)*x*_*k*_ *U*_1/*m*_*j*__(−*t*), where *j* ∈ {1,…, *l*} and *k* ∈ {1,…, *n*} is an important tool to study time decay of solutions to nonlinear Schrödinger equations satisfying the gauge invariant condition (6) since it acts as a differential operator. Fractional power of *J*_1/*m*_*j*__ is defined as

(18) $$\begin{array}{c} {\left|J_{1/m_{j}}\right|}^{a}\left(t\right)=U_{1/m_{j}}\left(t\right){\left|x\right|}^{a}U_{1/m_{j}}\left(-t\right),\;a>0, \end{array}$$

which is also represented as (see [18])

(19) $$\begin{array}{c} {\left|J_{1/m_{j}}\right|}^{a}\left(t\right)=M^{-m_{j}}{\left(-\frac{t^{2}}{m_{j}^{2}}\Delta \right)}^{a/2}M^{m_{j}}, \end{array}$$

for *t* ≠ 0. Moreover, for *L*_1/*m*_*j*__ = *i*∂_*t*_ + (1/2*m*_*j*_)Δ, we have commutation relations such that

(20) $$\begin{array}{c} \left[L_{1/m_{j}},{\left|J_{1/m_{j}}\right|}^{a}\right]=0. \end{array}$$

**Remark 1**

The system (1) includes some important nonlinear Schrödinger systems from the physical point of view. For example, the following system appears in a physical model (see e.g., [10, 19]):

(21) $$\begin{array}{c} i\partial_{t}v_{1}+\frac{1}{2m_{1}}\Delta v_{1}=-\left|v_{1}\right|v_{1}-\bar{v_{2}}v_{3},\\ i\partial_{t}v_{2}+\frac{1}{2m_{2}}\Delta v_{2}=-\left|v_{2}\right|v_{2}-v_{3}\bar{v_{1}},\\ i\partial_{t}v_{3}+\frac{1}{2m_{3}}\Delta v_{3}=-\left|v_{3}\right|v_{3}-v_{1}v_{2}, \end{array}$$

in (*t*, *x*) ∈ ℝ × ℝ^2^, where *m*_*j*_ is a mass of particle for *j* = 1,2, 3. In [10], the system (21) has been derived as a model describing nonlinear interactions between a laser beam and a plasma. In [19], the stability of solitary waves for the system (21) was investigated.

This paper is organized as follows.  Section 2 is devoted to present our recent works and some remarks. From Section 2.1 to Section 2.5, we consider the asymptotic behavior of solutions to nonlinear Schrödinger systems. In Section 2.1, we survey the results on the time decay estimates of solutions to nonlinear Schrödinger systems for *n* = 2 shown in [4–7, 9]. In Section 2.2, wave operators of nonlinear Schrödinger systems are investigated for *n* = 2 based on a paper [1].  Section 2.3 is concerned with the study of modified wave operators for *n* = 2 from a paper [1]. In the last two subsections, we survey the results in [2, 3]. In the last section we consider the related and open problems.

## 2. Nonlinear Schrödinger Systems

### 2.1. Time Decay of Solutions to Nonlinear Schrödinger Systems in Two Space Dimensions

To state time decay of solutions to nonlinear Schrödinger systems for *n* = 2, we start with time decay estimates of solutions to linear Schrödinger systems.

For (1), the corresponding linear system is written as

(22) $$\begin{array}{c} i\partial_{t}v_{j}+\frac{1}{2m_{j}}\Delta v_{j}=0,\;t\in \mathbb{R},\;\;x\in {\mathbb{R}}^{2},\\ v_{j}\left(0,x\right)=\phi_{j},\;x\in {\mathbb{R}}^{2}, \end{array}$$

where 1 ≤ *j* ≤ *l*.

As we know the solution *v*_*j*_(*t*) of (22) is represented as *v*_*j*_(*t*) = *U*_1/*m*_*j*__(*t*)*ϕ*_*j*_. It is known that *v*_*j*_(*t*) is decomposed into a main term and a remainder one as

(23) $$\begin{array}{c} U_{1/m_{j}}\left(t\right)\phi_{j}=e^{(im_{j}/2t){\left|x\right|}^{2}}\frac{m_{j}}{it}ℱ\phi_{j}\left(\frac{m_{j}x}{t}\right)+R_{j} \end{array}$$

for *n* = 2, where *R*_*j*_ decays rapidly in time; indeed we have the estimate

(24) $$\begin{array}{c} {||R_{j}||}_{L^{\infty }({\mathbb{R}}^{2})}\leq C{\left|t\right|}^{-1-(\gamma /2)}{||\phi_{j}||}_{{\dot{H}}^{0,1+\gamma }({\mathbb{R}}^{2})},\;0\leq \gamma \leq 1, \end{array}$$

for *t* ≠ 0. By ||*U*_1/*m*_*j*__(*t*)*v*_*j*_||_**L**^2^(ℝ^2^)_ = ||*v*_*j*_||_**L**^2^(ℝ^2^)_ and **L** ^*∞*^(ℝ^2^) − **L** ^1^(ℝ^2^) time decay estimate

(25) $$\begin{array}{c} {||U_{1/m_{j}}(t)v_{j}||}_{L^{\infty }({\mathbb{R}}^{2})}\leq {\left(\frac{2\pi }{m_{j}}\left|t\right|\right)}^{-1}{||v_{j}||}_{L^{1}({\mathbb{R}}^{2})} \end{array}$$

for *t* ≠ 0, we have the following time decay estimates through the interpolation theorem (see [12]).

**Theorem 2**

Let 2 ≤ *p* ≤ *∞*, and let *p*, *p*′ be conjugate indices, *t* ≠ 0. Then we have *U*_1/*m*_*j*__(*t*) : **L** ^*p*′^(ℝ^2^) → **L** ^*p*^(ℝ^2^) which are bounded operators and satisfy

(26) $$\begin{array}{c} {||U_{1/m_{j}}(t)v_{j}||}_{L^{p}({\mathbb{R}}^{2})}\leq {\left(\frac{2\pi }{m_{j}}\left|t\right|\right)}^{-2((1/2)-(1/p))}{||v_{j}||}_{L^{p^{\prime }}({\mathbb{R}}^{2})} \end{array}$$

for *j* = 1,…, *l*.

Now we consider a special type of the system (1),

(27) $$\begin{array}{c} i\partial_{t}u_{1}+\frac{1}{2m_{1}}\Delta u_{1}=\lambda \bar{u_{1}}u_{2},\\ i\partial_{t}u_{2}+\frac{1}{2m_{2}}\Delta u_{2}=\mu u_{1}^{2}, \end{array}$$

in (*t*, *x*) ∈ ℝ × ℝ^2^, where *m*_1_ and *m*_2_ are the masses of particles and *λ*, *μ* ∈ *ℂ*. If we let $u_{1}=\left(1/\sqrt{\left|\lambda \mu \right|}\right)v_{1}$ and *u*_2_ = (*μ*/|*λμ*|)*v*_2_ in the above system, then we obtain the system as below

(28) $$\begin{array}{c} i\partial_{t}v_{1}+\frac{1}{2m_{1}}\Delta v_{1}=\gamma \bar{v_{1}}v_{2},\\ i\partial_{t}v_{2}+\frac{1}{2m_{2}}\Delta v_{2}=v_{1}^{2}, \end{array}$$

in (*t*, *x*) ∈ ℝ × ℝ^2^, where *γ* = *λμ*/|*λμ*| ∈ *ℂ*. Therefore we survey the results on time decay of solutions to the system (28). The first result was obtained in [4].

**Theorem 3 (see [4])**

Assume that 2*m*_1_ = *m*_2_ and *γ* = 1. Then there exists *ɛ* > 0 such that (28) with the initial data

(29) $$\begin{array}{c} v\left(0\right)=\left(v_{1}\left(0\right),v_{2}\left(0\right)\right)=\left(\phi_{1},\phi_{2}\right)=\phi \end{array}$$

has a unique global solution

(30) $$\begin{array}{c} v=\left(v_{1},v_{2}\right)\in C\left(\mathbb{R};H^{2}\left({\mathbb{R}}^{2}\right)\cap H^{0,2}\left({\mathbb{R}}^{2}\right)\right) \end{array}$$

for any (*ϕ*_1_, *ϕ*_2_) ∈ **H** ^2^(ℝ^2^)∩**H** ^0,2^(ℝ^2^) satisfying

(31) $$\begin{array}{c} {||\phi ||}_{H^{2}({\mathbb{R}}^{2})\cap H^{0,2}({\mathbb{R}}^{2})}=\overset{2}{\underset{j=1}{\sum }}{||\phi_{j}||}_{H^{2}({\mathbb{R}}^{2})\cap H^{0,2}({\mathbb{R}}^{2})}\leq ɛ. \end{array}$$

Moreover the time decay estimate

(32) $$\begin{array}{c} {||v\left(t,\cdot \right)||}_{L^{\infty }({\mathbb{R}}^{2})}=\overset{2}{\underset{j=1}{\sum }}{||v_{j}\left(t,\cdot \right)||}_{L^{\infty }({\mathbb{R}}^{2})}\leq C{\left(1+\left|t\right|\right)}^{-1} \end{array}$$

is true for all *t* ∈ ℝ.

In Theorem 3, the main result is **L** ^*∞*^(ℝ^2^) time decay estimates of solutions of (28) and which is the same rate as that of the corresponding free solutions.

When *γ* = 1, (28) satisfies the condition (8). Under the condition, *γ* = 1, (28) obeys the **L** ^2^(ℝ^2^) conservation law such that

(33) $$\begin{array}{c} \frac{d}{dt}\left({||v_{1}||}_{L^{2}\left({\mathbb{R}}^{2}\right)}^{2}+{||v_{2}||}_{L^{2}\left({\mathbb{R}}^{2}\right)}^{2}\right)=0. \end{array}$$

In the case of the mass resonance condition 2*m*_1_ = *m*_2_, (28) satisfies the condition (6). Global existence of small solutions for (28) is obtained from the conservation law and the Strichartz estimate. **L** ^*∞*^(ℝ^2^) time decay of small solutions for (28) is proved through a priori estimates of local solutions in the norm ||*ℱU*_1/*m*_*j*__(−*t*)*v*_*j*_||_**L**^*∞*^(ℝ^2^)_. The similar idea has been used for construction of **H** ^2,2^(ℝ^2^) solutions to a single nonlinear Schrödinger equation by a paper [20]. Theorem 3 extends this idea to (28). The main point in the proof of the result is to derive the ordinary differential equation

(34) $$\begin{array}{c} i\partial_{t}\psi_{1}=\gamma t^{-1}\bar{\psi_{1}}\psi_{2}+O\left(t^{-1-ɛ}\right),\\ i\partial_{t}\psi_{2}=t^{-1}\psi_{1}^{2}+O\left(t^{-1-ɛ}\right), \end{array}$$

under the condition 2*m*_1_ = *m*_2_ by using the factorization formulas of Schrödinger evolution group stated in Section 1, where *ψ*_*j*_ = *D*_1/*m*_*j*__ *ℱU*_1/*m*_*j*__(−*t*)*v*_*j*_ for *j* = 1,2 and *ɛ* > 0. Asymptotic behavior in time of solutions of (28) is determined by that of the ordinary differential equations (34). The main task is to show that remainder terms are estimated from above by *O*(*t* ^−1−*ɛ*^) which is integrable in time. This is the reason why we use the condition such that the data must be in **H** ^0,*β*^(ℝ^2^), *β* > 1.

The system (1) is a generalization of (28). For the system (1), we have global existence theorem and time decay estimates as follows.

**Theorem 4 (see [5])**

One assumes that *ϕ* = (*ϕ*_1_,…, *ϕ*_*l*_) ∈ **H** ^2,2^(ℝ^2^) and *F*_*j*_ satisfies the conditions (6) and (8) for each *j* ∈ {1,…, *l*}. Then there exists *ɛ* > 0 such that (1) has a unique global solution

(35) $$\begin{array}{c} v=\left(v_{1},\ldots ,v_{l}\right)\in C\left(\mathbb{R};H^{2,2}\left({\mathbb{R}}^{2}\right)\right) \end{array}$$

for any *ϕ* = (*ϕ*_1_,…, *ϕ*_*l*_) ∈ **H** ^2,2^(ℝ^2^) satisfying

(36) $$\begin{array}{c} {||\phi ||}_{H^{2,2}({\mathbb{R}}^{2})}=\overset{l}{\underset{i=1}{\sum }}{||\phi_{i}||}_{H^{2,2}({\mathbb{R}}^{2})}\leq ɛ. \end{array}$$

Moreover the time decay estimate

(37) $$\begin{array}{c} {||v\left(t,\cdot \right)||}_{L^{\infty }({\mathbb{R}}^{2})}=\overset{l}{\underset{i=1}{\sum }}{||v_{i}\left(t,\cdot \right)||}_{L^{\infty }({\mathbb{R}}^{2})}\leq C{\left(1+\left|t\right|\right)}^{-1} \end{array}$$

is true for all *t* ∈ ℝ.

Theorem 4 was improved in [6] by replacing the condition such that *ϕ* ∈ **H** ^2,2^(ℝ^2^) by *ϕ* ∈ **H** ^*β*^(ℝ^2^)∩**H** ^0,*β*^(ℝ^2^) with 1 < *β*.

Now we focus on the following system:

(38) $$\begin{array}{c} i\partial_{t}v_{1}+\frac{1}{2m_{1}}\Delta v_{1}=\lambda_{1}\left|v_{1}\right|v_{1}+\mu_{1}\bar{v_{2}}v_{3},\\ i\partial_{t}v_{2}+\frac{1}{2m_{2}}\Delta v_{2}=\lambda_{2}\left|v_{2}\right|v_{2}+\mu_{2}\bar{v_{1}}v_{3},\\ i\partial_{t}v_{3}+\frac{1}{2m_{3}}\Delta v_{3}=\lambda_{3}\left|v_{3}\right|v_{3}+\mu_{3}v_{1}v_{2},\\ v_{j}\left(0,x\right)=\phi_{j}\left(x\right),\;j=1,2,3, \end{array}$$

in (*t*, *x*) ∈ ℝ × ℝ^2^, where *m*_1_, *m*_2_, *m*_3_ are the masses of particles and *λ*_1_, *λ*_2_, *λ*_3_, *μ*_1_, *μ*_2_, *μ*_3_ ∈ *ℂ*∖{0} are constants.

Time decay problem of solutions to (38) is considered in [7]. By using the similar method as [4, 5], we have global existence in time and time decay estimates of small solutions for (38) as below.

**Theorem 5 (see [7])**

Assume that the mass resonance condition *m*_1_ + *m*_2_ = *m*_3_ is satisfied. One also assumes that *Im*⁡*λ*_*j*_ ≤ 0 for *j* = 1,2, 3 and $\kappa_{1}\mu_{1}+\kappa_{2}\mu_{2}=\kappa_{3}\overset{-}{\mu_{3}}$ with some *κ*_1_, *κ*_2_, *κ*_3_ > 0. Then there exists *ɛ* > 0 such that (38) has a unique global solution

(39) $$\begin{array}{c} v=\left(v_{1},v_{2},v_{3}\right)\in C\left(\mathbb{R};H^{s}\left({\mathbb{R}}^{2}\right)\cap H^{0,s}\left({\mathbb{R}}^{2}\right)\right) \end{array}$$

for any *ϕ* = (*ϕ*_1_, *ϕ*_2_, *ϕ*_3_) ∈ **H** ^*s*^(ℝ^2^)∩**H** ^0,*s*^(ℝ^2^) satisfying

(40) $$\begin{array}{c} {||\phi ||}_{H^{s}({\mathbb{R}}^{2})\cap H^{0,s}({\mathbb{R}}^{2})}=\overset{3}{\underset{i=1}{\sum }}{||\phi_{i}||}_{H^{s}({\mathbb{R}}^{2})\cap H^{0,s}({\mathbb{R}}^{2})}\leq ɛ, \end{array}$$

where 1 < *s* < 2. Moreover, the time decay estimate

(41) $$\begin{array}{c} {||v(t,\cdot )||}_{L^{\infty }({\mathbb{R}}^{2})}=\overset{3}{\underset{i=1}{\sum }}{||v_{i}\left(t,\cdot \right)||}_{L^{\infty }({\mathbb{R}}^{2})}\leq C{\left(1+\left|t\right|\right)}^{-1} \end{array}$$

is true for all *t* ∈ ℝ.

If

(42) Im⁡λj<0 for  j=1,2,3,

the nonlinear term *λ*_*j*_ | *v*_*j*_ | *v*_*j*_ acts as a dissipation one which requires logarithmic correction in time of solutions and the negative time is not considered. We have the following theorem.

**Theorem 6 (see [7])**

Suppose that the assumptions of Theorem 5 are fulfilled. Let *v* be the solution to the system (38) constructed in Theorem 5. If

(43) Im⁡λj<0 for  j=1,2,3

is satisfied, then the time decay estimate

(44) $$\begin{array}{c} {||v(t,\cdot )||}_{L^{\infty }({\mathbb{R}}^{2})}\leq C{\left(1+t\right)}^{-1}{\left(\log\left(2+t\right)\right)}^{-1} \end{array}$$

is true for all *t* ≥ 0.

This phenomenon was found in [21] for a single equation. We note here that the method presented in [7] is different from the one in [21]. It seems that the proof in [21] does not work for the system.

Define the scaled function by *v*_*j*,*μ*_(*t*) = *μ* ^2^ *v*_*j*_(*μ* ^2^ *t*, *μx*); then *v*_*j*,*μ*_(*t*) satisfies the system (1) with the initial data *ϕ*_*j*,*μ*_(*x*) = *μ* ^2^ *ϕ*_*j*_(*μx*). We have

(45) ||ϕj,μ||H˙0,2−(n/2)(ℝn)2=∫ℝnμ4|x|4−n|ϕj(μx)|2dx=||ϕj||H˙0,2−(n/2)(ℝn)2,

which implies that ${\dot{H}}^{0,2-(n/2)}\left({\mathbb{R}}^{n}\right)$ is one of the so-called invariant spaces for the problem (1). Other invariant spaces of (1) are given by ${\dot{H}}^{(n/2)-2}\left({\mathbb{R}}^{n}\right)$. We consider the large time asymptotics of solutions to the system (1) for *n* = 2 in the function space ${\dot{H}}^{0,\alpha }\left({\mathbb{R}}^{2}\right)\cap {\dot{H}}^{0,\beta }\left({\mathbb{R}}^{2}\right)$, where *α*, *β* satisfy 0 ≤ *β* < 1 < *α* ≤ 2 and can be taken to be close to 1. Therefore our function space has relation with the invariant space and the considered data are not necessarily in **L** ^2^(ℝ^2^).

**Theorem 7 (see [9])**

Assume that (6) and (8) hold. One also assumes that $\phi =\left(\phi_{1},\ldots ,\phi_{l}\right)\in {\dot{H}}^{0,\alpha }\left({\mathbb{R}}^{2}\right)\cap {\dot{H}}^{0,\beta }\left({\mathbb{R}}^{2}\right)$, where 0 ≤ *β* < 1 < *α* ≤ 2. Then there exists *ɛ* > 0 such that (1) has a unique global solution *v* such that

(46) U1/m(−t)v=(U1/m1(−t)v1,…,U1/ml(−t)vl)∈C(ℝ;H˙0,α(ℝ2)∩H˙0,β(ℝ2))

for any *ϕ* = (*ϕ*_1_,…, *ϕ*_*l*_) satisfying

(47) $$\begin{array}{c} {||\phi ||}_{{\dot{H}}^{0,\alpha }({\mathbb{R}}^{2})\cap {\dot{H}}^{0,\beta }({\mathbb{R}}^{2})}=\overset{l}{\underset{i=1}{\sum }}{||\phi_{i}||}_{{\dot{H}}^{0,\alpha }({\mathbb{R}}^{2})\cap {\dot{H}}^{0,\beta }({\mathbb{R}}^{2})}\leq ɛ. \end{array}$$

Moreover, the time decay estimate

(48) $$\begin{array}{c} {||v\left(t,\cdot \right)||}_{L^{\infty }({\mathbb{R}}^{2})}=\overset{l}{\underset{i=1}{\sum }}{||v_{i}\left(t,\cdot \right)||}_{L^{\infty }({\mathbb{R}}^{2})}\leq C{\left|t\right|}^{-1} \end{array}$$

is true for *t* ≠ 0.

We note that in [8] we consider the problem (38) under the initial condition $\phi =(\phi_{1},\phi_{2}{,\phi }_{3})\in {\dot{H}}^{0,\alpha }\left({\mathbb{R}}^{2}\right)\cap {\dot{H}}^{0,\delta }\left({\mathbb{R}}^{2}\right)$ and the dissipation condition *Im*⁡*λ*_*j*_ < 0, where 0 ≤ *δ* < 1 < *α* ≤ 2. Then we have the time decay estimate such that

(49) $$\begin{array}{c} {||v(t,\cdot )||}_{L^{\infty }({\mathbb{R}}^{2})}\leq t^{-1}{\left(\log t\right)}^{-1} \end{array}$$

for all *t* ≥ 1.

In the final part of this subsection, we will show that the assumption (8) is important for obtaining the time decay estimates of solutions. Let us consider the following system:

(50) $$\begin{array}{c} i\partial_{t}v_{1}+\frac{1}{2m_{1}}\Delta v_{1}=0,\\ i\partial_{t}v_{2}+\frac{1}{2m_{2}}\Delta v_{2}=v_{1}^{2},\\ v_{1}\left(0\right)=\phi_{1},\;v_{2}\left(0\right)=\phi_{2}, \end{array}$$

in (*t*, *x*) ∈ ℝ × ℝ^2^, where *m*_1_, *m*_2_ are the masses of particles. It is obvious that the nonlinearities of (50) do not satisfy the assumption (8). Since the first equation of this system with the initial condition *v*_1_(0) = *ϕ*_1_ can be considered the Cauchy problem for the linear Schrödinger equation, we find the value of *v*_1_ explicitly by *v*_1_ = *U*_1/*m*_1__(*t*)*ϕ*_1_. Therefore, we have

(51) $$\begin{array}{c} i\partial_{t}v_{2}+\frac{1}{2m_{2}}\Delta v_{2}={\left(U_{1/m_{1}}\left(t\right)\phi_{1}\right)}^{2},\\ v_{2}\left(0\right)=\phi_{2}. \end{array}$$

For the system (51), we obtain the following result.

**Proposition 8 (see [4])**

Suppose that *ϕ*_1_ ∈ **H** ^0,2^(ℝ^2^),  *ϕ*_2_ ∈ **L** ^2^(ℝ^2^). Let

(52) $$\begin{array}{c} v_{2}\in C\left(\left[1,\infty \right);L^{2}\left({\mathbb{R}}^{2}\right)\right) \end{array}$$

be a global solution of (51). Then the following estimate is true:

(53) $$\begin{array}{c} {||v_{2}\left(t\right)||}_{L^{2}({\mathbb{R}}^{2})}\geq m_{1}{||\hat{\phi_{1}}||}_{L^{4}({\mathbb{R}}^{2})}^{2}\log t-C{||\phi_{1}||}_{H^{0,2}({\mathbb{R}}^{2})}^{2} \end{array}$$

for *t* > 1.

This fact was pointed first in [22, 23] in the case of Klein-Gordon equations and in Remark 3 of [24] in the case of Schrödinger equations.

### 2.2. Wave Operators of Nonlinear Schrödinger Systems in Two Space Dimensions

First, we briefly explain the definition of the wave operator (see [25]). For a given function *v*_+_ = (*v*_*i*+_), we assume that there exists a unique solution *v* = (*v*_*i*_) of the system (1) satisfying the asymptotics

(54) $$\begin{array}{c} \underset{t\to \infty }{\lim}{||v(t)-U_{1/m}(t)v_{+}||}_{X}=0, \end{array}$$

where *U*_1/*m*_(*t*)*v*_+_ is the solution of linear problems with the initial data *v*_+_ and ||·||_*X*_ is the norm of Banach space *X*. Then we define the map *W*_+_ : *v*_+_ ↦ *v*(*t*) and call it the wave operator. We also call *v*_+_ the final state (or the final value) since it is considered the value of *U*_1/*m*_(−*t*)*v*(*t*) at infinity if *U*_1/*m*_(*t*) is the unitary operator in *X*. The same problem can be considered for negative time.

To study existence of wave operators for (28), we consider the following problem for given final data (*ϕ*_1+_, *ϕ*_2+_):

(55) $$\begin{array}{c} i\partial_{t}v_{1}+\frac{1}{2m_{1}}\Delta v_{1}=\gamma \bar{v_{1}}v_{2},\\ i\partial_{t}v_{2}+\frac{1}{2m_{2}}\Delta v_{2}=v_{1}^{2},\\ {||v_{1}\left(t\right)-U_{1/m_{1}}\left(t\right)\phi_{1+}||}_{L^{2}({\mathbb{R}}^{2})}⟶0\;\text{as}\;t⟶\infty ,\\ {||v_{2}\left(t\right)-U_{1/m_{2}}\left(t\right)\phi_{2+}||}_{L^{2}({\mathbb{R}}^{2})}⟶0\;\text{as}\;t⟶\infty , \end{array}$$

in (*t*, *x*) ∈ ℝ × ℝ^2^. If there exists a nontrivial solution for the above system, then we say that there exists a usual wave operator.

We consider (28) under the mass nonresonance conditions 2*m*_1_ ≠ *m*_2_ and *m*_1_ ≠ *m*_2_.

**Theorem 9 (see [1])**

Let 2*m*_1_ ≠ *m*_2_ and *m*_1_ ≠ *m*_2_. Then there exists *ɛ* > 0 such that, for any

(56) $$\begin{array}{c} \phi_{+}=\left(\phi_{1+},\phi_{2+}\right)\in \left(H^{0,2}\left({\mathbb{R}}^{2}\right)\cap {\dot{H}}^{-2b}\left({\mathbb{R}}^{2}\right)\right)\times H^{0,2}\left({\mathbb{R}}^{2}\right) \end{array}$$

with the norm

(57) $$\begin{array}{c} {||\phi_{1+}||}_{H^{0,2}({\mathbb{R}}^{2})\cap {\dot{H}}^{-2b}({\mathbb{R}}^{2})}+{||\phi_{2+}||}_{H^{0,2}({\mathbb{R}}^{2})}\leq ɛ, \end{array}$$

the system (28) has a unique global solution

(58) $$\begin{array}{c} v=\left(v_{1},v_{2}\right)\in C\left(\left[1,\infty \right);L^{2}\left({\mathbb{R}}^{2}\right)\right). \end{array}$$

Moreover, the following estimate

(59) ||v(t)−U1/m(t)ϕ+||L2(ℝ2) =∑j=12||vj(t)−U1/mj(t)ϕj+||L2(ℝ2)≤Ct−b

holds for all *t* ≥ 1, where 1/2 < *b* < 1.

The mass nonresonance conditions 2*m*_1_ ≠ *m*_2_ and *m*_1_ ≠ *m*_2_ are used to obtain better time decay of solutions. Oscillating properties of nonlinear terms $\bar{v_{1+}}v_{2+}$ and *v*_1+_^2^ are different from those of solutions to linear problem which yield an additional time decay from nonlinear terms; namely, nonlinear interactions are not critical. By combining this fact and the Strichartz type estimates, the result of Theorem 9 is obtained.

We next consider (28) under the mass condition *m*_1_ = *m*_2_ which is also the mass nonresonance case and the support conditions on the data.

**Theorem 10 (see [1])**

Let *m*_1_ = *m*_2_. Assume that

(60) $$\begin{array}{c} \phi_{+}=\left(\phi_{1+},\phi_{2+}\right)\in \left(H^{0,2}\left({\mathbb{R}}^{2}\right)\cap {\dot{H}}^{-2b,0}\left({\mathbb{R}}^{2}\right)\right)\times H^{1/2,2}\left({\mathbb{R}}^{2}\right),\\ \mathrm{supp}\;\hat{\phi_{1+}}\cap \mathrm{supp}\;\hat{\phi_{2+}}\;is\;\;empty. \end{array}$$

Then there exists *ɛ* > 0 such that, for any *ϕ*_+_ = (*ϕ*_1+_, *ϕ*_2+_) with the norm

(61) $$\begin{array}{c} {||\phi_{1+}||}_{H^{0,2}({\mathbb{R}}^{2})\cap {\dot{H}}^{-2b,0}({\mathbb{R}}^{2})}+{||\phi_{2+}||}_{H^{0,2}({\mathbb{R}}^{2})}\leq ɛ, \end{array}$$

there exists a unique solution

(62) $$\begin{array}{c} v=\left(v_{1},v_{2}\right)\in C\left(\left[1,\infty \right);L^{2}\left({\mathbb{R}}^{2}\right)\right) \end{array}$$

for the system (28) satisfying the estimate

(63) ||v(t)−U1/m(t)ϕ+||L2(ℝ2) =∑j=12||vj(t)−U1/mj(t)ϕj+||L2(ℝ2)≤Ct−b

for all *t* ≥ 1, where 1/2 < *b* < 3/4.

From the result we have wave operators when the support of the Fourier transform of the Schrödinger data is restricted. Restriction on the support of the Fourier transform of the Schrödinger data was used to obtain an improved time decay estimate of the nonlinear term $\bar{v_{1+}}v_{2+}$.

We turn to investigate existence of wave operators for (1). First, we give a necessary condition of existence of asymptotically free solutions.

**Theorem 11 (see [5])**

Let *ϕ* = (*ϕ*_1_,…, *ϕ*_*l*_) ∈ **H** ^2,2^(ℝ^2^) and let *v* be global in time of solutions of (1) satisfying a priori estimates

(64) ∫1∞1s2||U1/m(−s)v||H0,2(ℝ2)2ds<∞, ||ℱU1/m(−t)v||L∞(ℝ2)≤C.

We assume that the gauge condition (6) holds for each *j* ∈ {1,…, *l*}. If there exists $\hat{\psi_{+}}=\left(\hat{\psi_{1+}},\ldots ,\hat{\psi_{l+}}\right)\in L^{2}\left({\mathbb{R}}^{2}\right)\cap L^{\infty }\left({\mathbb{R}}^{2}\right)$ such that

(65) $$\begin{array}{c} \underset{t\to \infty }{\lim}{||v\left(t\right)-U_{1/m}\left(t\right)\psi_{+}||}_{L^{2}({\mathbb{R}}^{2})}=0, \end{array}$$

then

(66) $$\begin{array}{c} F_{j}\left(D_{1/m_{1}}\hat{\psi_{1+}},\ldots ,D_{1/m_{l}}\hat{\psi_{l+}}\right)=0 \end{array}$$

for every *j* ∈ {1,…, *l*}, where $\hat{\psi_{+}}=ℱ\psi_{+}$.

If the support condition

(67) $$\begin{array}{c} \overset{l}{\underset{j=1}{⋂}}\mathrm{supp}\;\hat{\psi_{j+}}\left(m_{j}\xi \right)\;\text{is}\;\text{empty} \end{array}$$

is satisfied, we have (66).

We give existence of wave operators of the system (1) for small final states by (66).

**Theorem 12 (see [5])**

Let $\hat{\psi_{+}}=\left(\hat{\psi_{1+}},\ldots ,\hat{\psi_{l+}}\right)\in H^{2,2}\left({\mathbb{R}}^{2}\right)$ satisfy the so-called support condition (66). Assume that *F*_*j*_ satisfies the gauge condition (6) for each *j* ∈ {1,…, *l*}. Then for some *ɛ* > 0 there exists a unique global solution *v* = (*v*_1_,…, *v*_*l*_) of the system (1) such that

(68) $$\begin{array}{c} v\in C\left(\left[1,\infty \right);L^{2}\left({\mathbb{R}}^{2}\right)\right),\\ {||v\left(t\right)-U_{1/m}\left(t\right)\psi_{+}||}_{L^{2}({\mathbb{R}}^{2})}\leq Ct^{-b},\;1/2<b<1 \end{array}$$

for large *t* and any $\hat{\psi_{+}}$ satisfying

(69) $$\begin{array}{c} {||\psi_{+}||}_{H^{2,2}({\mathbb{R}}^{2})}\leq ɛ. \end{array}$$

Existence of wave operator for a single nonlinear Schrödinger equation was studied in [26, 27].

### 2.3. Modified Wave Operators of Nonlinear Schrödinger Systems in Two Space Dimensions

In Section 2.2, we discuss the existence of wave operators of the systems (1) and (28). However, if it is impossible to show existence of the wave operator, we have to modify the setting of the problem. We define the modified wave operator (see [25]) as follows. Let us construct a function *v*_+_ = (*v*_*i*+_) from a suitable function space and define a function *f*(*v*_+_, *t*) by *v*_+_. Then we try to find a unique solution of nonlinear problems under the asymptotic condition

(70) $$\begin{array}{c} \underset{t\to \infty }{\lim}{||v(t)-U_{1/m}(t)f(v_{+},t)||}_{X}=0, \end{array}$$

where ||·||_*X*_ is the norm of Banach space *X*. Namely, the problem is solved if we can define the function *f* satisfying the asymptotic condition (70) by taking the structure of nonlinear terms into consideration. If we have a positive answer, we can define the map *MW*_+_ : *v*_+_ ↦ *v*(*t*) instead of the wave operator. We call *MW*_+_ the modified wave operator since we modified the final states.

We consider (28) again which is written as

(71) $$\begin{array}{c} i\partial_{t}v_{1}+\frac{1}{2m_{1}}\Delta v_{1}=\gamma \bar{v_{1}}v_{2},\\ i\partial_{t}v_{2}+\frac{1}{2m_{2}}\Delta v_{2}=v_{1}^{2}, \end{array}$$

in (*t*, *x*) ∈ ℝ × ℝ^2^. In Section 2.1, we stated the time decay estimates of solutions to this system in the case of 2*m*_1_ = *m*_2_ and *γ* = 1. Since the nonlinearity is critical in this case, it is impossible to find a solution in the neighborhood of the free final state (*U*_1/*m*_1__(*t*)*ϕ*_1+_, *U*_1/*m*_2__(*t*)*ϕ*_2+_). Indeed we have the nonexistence of the usual scattering states.

**Theorem 13 (see [4])**

Let 2*m*_1_ = *m*_2_, *γ* = 1, and let

(72) $$\begin{array}{c} v=\left(v_{1},v_{2}\right)\in C\left(\left[0,\infty \right);H^{2}\left({\mathbb{R}}^{2}\right)\cap H^{0,2}\left({\mathbb{R}}^{2}\right)\right) \end{array}$$

be a global solution obtained in Theorem 3. Then there does not exist any nontrivial scattering state *ϕ*_+_ = (*ϕ*_1+_, *ϕ*_2+_) ∈ **H** ^2^(ℝ^2^)∩**H** ^0,2^(ℝ^2^) such that *ϕ*_1+_ ≠ 0 and

(73) $$\begin{array}{c} {||v\left(t\right)-U_{1/m}\phi_{+}||}_{L^{2}({\mathbb{R}}^{2})}=\overset{2}{\underset{j=1}{\sum }}{||v_{j}\left(t\right)-U_{1/m_{j}}\phi_{j+}||}_{L^{2}({\mathbb{R}}^{2})}⟶0 \end{array}$$

as *t* → *∞*.

From the result, we need to modify the final state with time dependence. We note here that the modified wave operator for nonlinear dispersive equation was first constructed in [28] for the cubic nonlinear Schrödinger equations in one space dimension and then constructed in [29] for the derivative nonlinear Schrödinger equation, by changing it via a suitable transformation (see [30]) to a system of cubic nonlinear Schrödinger equations without derivatives of unknown function; see also [31] for recent developments. Two-dimensional case was studied in [32].

In Section 2.2, from (34), we see that the asymptotic behavior of solutions of (28) under the mass resonance condition 2*m*_1_ = *m*_2_ is determined by the solutions of the following system:

(74) $$\begin{array}{c} i\partial_{t}\varphi_{1\gamma }=\gamma t^{-1}\bar{\varphi_{1\gamma }}\varphi_{2\gamma },\\ i\partial_{t}\varphi_{2\gamma }=t^{-1}\varphi_{1\gamma }^{2}. \end{array}$$

By calculation we find that the particular solutions of (74) are

(75) $$\begin{array}{c} \varphi_{1\gamma }\left(t,\xi \right)=-\frac{i\omega \left(\xi \right)e^{i\theta (\xi )}}{1+\omega \left(\xi \right)\log t},\\ \varphi_{2\gamma }\left(t,\xi \right)=-\frac{i\omega \left(\xi \right)e^{2i\theta (\xi )}}{1+\omega \left(\xi \right)\log t} \end{array}$$

in the case of *γ* = −1 and the particular solutions of (74) are

(76) $$\begin{array}{c} \varphi_{1\gamma }\left(t,\xi \right)=\omega \left(\xi \right)e^{i\theta (\xi )+(i/\sqrt{2})\omega (\xi )\log t}\\ \varphi_{2\gamma }\left(t,\xi \right)=-\frac{1}{\sqrt{2}}\omega \left(\xi \right)e^{2i\theta (\xi )+i\sqrt{2}\omega (\xi )\log t} \end{array}$$

in the case of *γ* = 1, where *ω*(*ξ*) > 0 and *θ*(*ξ*) is a real valued given function. We also find that

(77) $$\begin{array}{c} \varphi_{1\gamma }\left(\xi \right)=0,\;\;\varphi_{2\gamma }\left(\xi \right)=\omega \left(\xi \right)e^{i\theta (\xi )} \end{array}$$

are particular solutions of (74) when *γ* = ±1.

The following theorem shows existence of the modified wave operators of (28).

**Theorem 14 (see [1])**

Let 2*m*_1_ = *m*_2_ and *γ* = ±1. Then there exists *ɛ* > 0 such that, for any *ω*, *θ* ∈ **H** ^2^(ℝ^2^) with norm ||*ω*||_**H**^2^(ℝ^2^)_ ≤ *ɛ*, (28) has a unique global solution

(78) $$\begin{array}{c} v=\left(v_{1},v_{2}\right)\in C\left(\left[1,\infty \right);L^{2}\left({\mathbb{R}}^{2}\right)\right). \end{array}$$

Moreover, the following estimate

(79) ||v(t)+U1/m(t)ℱ−1Dmφγ(t)||L2(ℝ2) =∑j=12||vj(t)+U1/mj(t)ℱ−1Dmjφjγ(t)||L2(ℝ2)≤Ct−b

holds for all *t* ≥ 1, where 1/2 < *b* < 1.

Using the resonance condition, 2*m*_1_ = *m*_2_, we get the existence of modified wave operators by the contraction mapping principle. Since the identity *U*_1/*m*_*j*__(*t*) = *M* ^−*m*_*j*_^ *D*_*t*/*m*_*j*__ *ℱM* ^−*m*_*j*_^ is known for *j* = 1,2, we have the estimate from the above theorem

(80) $$\begin{array}{c} {||v\left(t\right)-iM^{-m}D_{t}ℱM^{-1/m}{ℱ}^{-1}\varphi_{\gamma }\left(t\right)||}_{L^{2}({\mathbb{R}}^{2})}\leq Ct^{-b} \end{array}$$

for all *t* ≥ 1, where 1/2 < *b* < 1.

Existence of modified wave operator for a single nonlinear Schrödinger equation was studied in [26, 27]. Asymptotic behavior of solutions to nonlinear wave systems was studied in [33]. It was shown that the asymptotic behavior of solutions of them depends on the corresponding ordinary differential equations which are related to (74). In [33], another special solution was presented and the method can be applicable to nonlinear Schrödinger systems with a slight modification.

### 2.4. Nonlinear Schrödinger Systems in Higher Space Dimensions I

In the case of higher space dimensions, *n* ≥ 3, the scattering theory for (28)

(81) $$\begin{array}{c} i\partial_{t}v_{1}+\frac{1}{2m_{1}}\Delta v_{1}=\gamma \bar{v_{1}}v_{2},\\ i\partial_{t}v_{2}+\frac{1}{2m_{2}}\Delta v_{2}=v_{1}^{2} \end{array}$$

was studied in [2].

We will explain the scattering problem (See [25]) briefly. We may assume the existence of wave operator *W*_+_ which maps a Banach space *X* into itself. Namely, for any given *v*_+_ ∈ *X*, we assume that there exists a unique solution *v*(*t*) ∈ **C**([0, *∞*); *X*) of the nonlinear system such that

(82) $$\begin{array}{c} \underset{t\to \infty }{\lim}{||U_{1/m}(-t)v(t)-v_{+}||}_{X}=0. \end{array}$$

We consider the initial value problem with the data *v*(0) which are determined by the solution *v*(*t*) in the time interval *t* ∈ [0, *∞*). If the initial value problem has a unique global solution *v*(*t*) ∈ **C**((−*∞*, 0]; *X*) and we can find a unique *v*_−_ ∈ *X* from the solution *v*(*t*) satisfying

(83) $$\begin{array}{c} \underset{t\to -\infty }{\lim}{||U_{1/m}(-t)v(t)-v_{-}||}_{X}=0, \end{array}$$

then we can define the inverse wave operator *W*_−_^−1^ : *v*(0) ∈ *X* ↦ *v*_−_ ∈ *X*. From this operator we can define *S* = *W*_−_^−1^ *W*_+_ : *X* → *X*. We call the operator the scattering operator.

In the case, *n* ≥ 4, existence of the scattering operator was proved in the space **H** ^(*n*/2)−2^(ℝ^*n*^) which is close to the invariant space ${\dot{H}}^{(n/2)-2}\left({\mathbb{R}}^{n}\right)$. In the case of *n* = 4, we have the results in the invariant space **L** ^2^(ℝ^4^). In the case of *n* = 3, existence of the scattering operator was proved in the space **H** ^0,1/2^(ℝ^3^), under the mass resonance condition 2*m*_1_ = *m*_2_, which is close to the invariant space ${\dot{H}}^{0,1/2}\left({\mathbb{R}}^{3}\right)$. To state the following theorem, we introduce

(84) Bɛ= {ϕ=(ϕ1,ϕ2)∈H(n/2)−2(ℝn);   ||ϕ||H(n/2)−2(ℝn)=∑j=12||ϕj||H(n/2)−2(ℝn)≤ɛ}.

**Theorem 15 (see [2])**

Let *n* ≥ 4. Then there exist *ε*_0_ and *C*_0_ such that 0 < *ε*_0_ ≤ 1 ≤ *C*_0_ with the following property.

(1) For any *ɛ* with 0 < *ɛ* ≤ *ε*_0_ and any *ϕ* = (*ϕ*_1_, *ϕ*_2_) ∈ *B*_*ɛ*_, (28) has a unique global solution *v* = (*v*_1_, *v*_2_) ∈ **C**(ℝ; **H** ^(*n*/2)−2^(ℝ^*n*^)). Moreover, there exist unique *ϕ*_±_ = (*ϕ*_1±_, *ϕ*_2±_) ∈ *B*_*C*_0_*ɛ*_ such that

(85) $$\begin{array}{c} {||v\left(t\right)-U_{1/m}\left(t\right)\phi_{\pm }||}_{H^{(n/2)-2}({\mathbb{R}}^{n})}⟶0 \end{array}$$

as *t* → ±*∞*.

(2)_+_ For any *ɛ* with 0 < *ɛ* ≤ *ε*_0_ and any *ϕ*_+_ = (*ϕ*_1+_, *ϕ*_2+_) ∈ *B*_*ɛ*_, (28) has a unique global solution *v* = (*v*_1_, *v*_2_) ∈ **C**(ℝ; **H** ^(*n*/2)−2^(ℝ^*n*^)) such that *v*(0) = (*v*_1_(0), *v*_2_(0)) ∈ *B*_*C*_0_*ɛ*_,

(86) $$\begin{array}{c} {||v\left(t\right)-U_{1/m}\left(t\right)\phi_{+}||}_{H^{(n/2)-2}({\mathbb{R}}^{n})}⟶0, \end{array}$$

as *t* → +*∞*.

(2)_−_ For any *ɛ* with 0 < *ɛ* ≤ *ε*_0_ and any *ϕ*_−_ = (*ϕ*_1−_, *ϕ*_2−_) ∈ *B*_*ɛ*_, (28) has a unique solution *v* = (*v*_1_, *v*_2_) ∈ **C**(ℝ; **H** ^(*n*/2)−2^(ℝ^*n*^)) such that *v*(0) = (*v*_1_(0), *v*_2_(0)) ∈ *B*_*C*_0_*ɛ*_,

(87) $$\begin{array}{c} {||v\left(t\right)-U_{1/m}\left(t\right)\phi_{-}||}_{H^{(n/2)-2}({\mathbb{R}}^{n})}⟶0 \end{array}$$

as *t* → −*∞*.

**Corollary 16 (see [2])**

The wave operators *W*_±_ : *ϕ*_±_ ↦ *v*(0) are defined as mappings from *B*_*ɛ*_ to *B*_*C*_0_*ɛ*_  for any *ɛ* with 0 < *ɛ* ≤ *ε*_0_. The scattering operator *S* : *ϕ*_+_ ↦ *ϕ*_−_ is defined as a mapping from *B*_*C*_0_^−1^*ɛ*_ to *B*_*C*_0_*ɛ*_ for any *ɛ* with 0 < *ɛ* ≤ *ε*_0_.To state the following theorem, we introduce

(88) B~ɛ=  {ϕ=(ϕ1,ϕ2)∈H0,1/2(ℝ3);   ||ϕ||H0,1/2(ℝ3)=∑j=12||ϕj||H0,1/2(ℝ3)≤ɛ}.

**Theorem 17 (see [2])**

Let  2*m*_1_ = *m*_2_. Then there exist *ε*_0_ and *C*_0_ such that 0 < *ε*_0_ ≤ 1 ≤ *C*_0_ with the following property.

(1) For any *ɛ* with 0 < *ɛ* ≤ *ε*_0_ and any $\phi \in {\tilde{B}}_{ɛ}$, (28) has a unique solution *v* with *U*_1/*m*_(−*t*)*v* ∈ **C**(ℝ; **H** ^0,1/2^(ℝ^3^)). Moreover, there exist unique $\phi_{\pm }\in {\tilde{B}}_{C_{0}ɛ}$ such that

(89) $$\begin{array}{c} {||U_{1/m}\left(-t\right)v\left(t\right)-\phi_{\pm }||}_{H^{0,1/2}({\mathbb{R}}^{3})}⟶0 \end{array}$$

as *t* → ±*∞*.

(2)_+_ For any *ɛ* with 0 < *ɛ* ≤ *ε*_0_ and any $\phi_{+}\in {\tilde{B}}_{ɛ}$, (28) has a unique solution *v* with *U*_1/*m*_(−*t*)*v* ∈ **C**(ℝ; **H** ^0,1/2^(ℝ^3^)) such that $v\left(0\right)\in {\tilde{B}}_{C_{0}ɛ}$,

(90) $$\begin{array}{c} {||U_{1/m}\left(-t\right)v\left(t\right)-\phi_{+}||}_{H^{0,1/2}({\mathbb{R}}^{3})}⟶0 \end{array}$$

as *t* → +*∞*.

(2)_−_ For any *ɛ* with 0 < *ɛ* ≤ *ε*_0_ and any $\phi_{-}\in {\tilde{B}}_{ɛ}$, (28) has a unique solution *v* with *U*_1/*m*_(−*t*)*v* ∈ **C**(ℝ; **H** ^0,1/2^(ℝ^3^)) such that $v\left(0\right)\in {\tilde{B}}_{C_{0}ɛ}$,

(91) $$\begin{array}{c} {||U_{1/m}\left(-t\right)v\left(t\right)-\phi_{-}||}_{H^{0,1/2}({\mathbb{R}}^{3})}⟶0 \end{array}$$

as *t* → −*∞*.

**Corollary 18 (see [2])**

The wave operators *W*_±_ : *ϕ*_±_ ↦ *v*(0) are defined as mappings from ${\tilde{B}}_{ɛ}$ to ${\tilde{B}}_{C_{0}ɛ}$  for any *ɛ* with 0 < *ɛ* ≤ *ε*_0_. The scattering operator *S* : *ϕ*_+_ ↦ *ϕ*_−_ is defined as a mapping from ${\tilde{B}}_{C_{0}^{-1}ɛ}$ to ${\tilde{B}}_{C_{0}ɛ}$ for any *ɛ* with 0 < *ɛ* ≤ *ε*_0_.

### 2.5. Nonlinear Schrödinger Systems in Higher Space Dimensions II

In [3], the finite time blow-up of the negative energy solutions for the system (28) was discussed in the case of 4 ≤ *n* ≤ 6 under the mass conditions 2*m*_1_ = *m*_2_ and *γ* ∈ ℝ. To state the blow-up result we need local existence in time of solutions to (28).

**Theorem 19 (see [3])**

Let *n* ≤ 6 and *m*_2_ = 2*m*_1_. Then, for any

(92) $$\begin{array}{c} \phi =\left(\phi_{1},\phi_{2}\right)\in H^{0,1}\left({\mathbb{R}}^{n}\right)\cap H^{1}\left({\mathbb{R}}^{n}\right), \end{array}$$

there exists *T*(*ϕ*) > 0 such that (28) has a unique solution *v* with

(93) U1/m(−t)v(t)=(U1/m1(−t)v1(t),U1/m2(−t)v2(t))∈C([−T,T];H0,1(ℝn)∩H1(ℝn)).

From Theorem 19 we have the energy conservation law such that

(94) 12m1||∇v1(t)||2+γ4m2||∇v2(t)||2+γRe(v2(t),v1(t)2) =12m1||∇ϕ1||2+γ4m2||∇ϕ2||2+γRe(ϕ2,ϕ12),

where

(95) $$\begin{array}{c} \left(f,g\right)=\int f\cdot \bar{g}\;dx. \end{array}$$

We need the virial identity to prove the blow-up result.

**Theorem 20 (see [3])**

Let *n* ≤ 6 and *m*_2_ = 2*m*_1_. Let *γ* ∈ ℝ and let *v* be the local solution constructed in Theorem 19. Then

(96) ||xv1(t)||L2(ℝn)2+γ||xv2(t)||L2(ℝn)2 =||xϕ1||L2(ℝn)2+γ||xϕ2||L2(ℝn)2+P0t+n2m1E0t2  +4−nm1∫0t(t−s)(12m1||∇v1(s)||L2(ℝn)2 +γ8m1||∇v2(s)||L2(ℝn)2)ds

for all *t* ∈ [−*T*, *T*], where

(97) P0  =2m1Im⁡(∇ϕ1,xϕ1)+γm1Im⁡(∇ϕ2,xϕ2),E0  =12m1||∇ϕ1||L2(ℝn)2+γ8m1||∇ϕ2||L2(ℝn)2+γRe(ϕ2,ϕ12).

By a standard argument, we have the following result

**Theorem 21 (see [3])**

Let 4 ≤ *n* ≤ 6. Let *m*_2_ = 2*m*_1_ and *γ* > 0. Let *ϕ* and *v* be as in Theorem 20. Then the maximal existence time for *v* is finite in the following cases:

(98) $$\begin{array}{c} E_{0}<0,\\ E_{0}=0,\;P_{0}<0, \end{array}$$

where *E*_0_ and *P*_0_ are as in Theorem 20.

## 3. Related and Open Problems

Asymptotic behavior in time of solutions to (28)

(99) $$\begin{array}{c} i\partial_{t}v_{1}+\frac{1}{2m_{1}}\Delta v_{1}=\gamma \bar{v_{1}}v_{2},\\ i\partial_{t}v_{2}+\frac{1}{2m_{2}}\Delta v_{2}=v_{1}^{2}, \end{array}$$

is an open problem for one space dimension which is considered the subcritical case. Existence of scattering operator is also an open problem for *n* = 1,2. We now turn to the relativistic version of (28)

(100) $$\begin{array}{c} \partial_{t}^{2}u_{1}-c^{2}\Delta u_{1}+m_{1}^{2}c^{4}u_{1}=-2\gamma c^{2}m_{1}u_{1}u_{2},\\ \partial_{t}^{2}u_{2}-c^{2}\Delta u_{2}+m_{2}^{2}c^{4}u_{2}=-2c^{2}m_{2}u_{1}^{2}. \end{array}$$

We let *c* = 1 in (100); then

(101) $$\begin{array}{c} \partial_{t}^{2}u_{1}-\Delta u_{1}+m_{1}^{2}u_{1}=\lambda_{1}u_{1}u_{2},\\ \partial_{t}^{2}u_{2}-\Delta u_{2}+m_{2}^{2}u_{2}=\lambda_{2}u_{1}^{2}, \end{array}$$

where *λ*_1_, *λ*_2_ ∈ *ℂ*. Asymptotic behavior of solutions to (101) when *n* = 2 was studied in papers [22, 34, 35] with mass nonresonance condition 2*m*_1_ ≠ *m*_2_. Scattering operator was constructed in a paper [36] if *m*_2_ < 2*m*_1_. However existence of scattering operator for (28) is an open problem even if *m*_2_ < 2*m*_1_. Global existence and time decay of small solutions were obtained in [37] for the resonance case 2*m*_1_ = *m*_2_, under some regularity and compactness conditions on the initial data. However the large time asymptotics and existence of modified scattering operators are not known for the case. The asymptotic behavior of solutions to (101) in one space dimension is also an open problem.

Small data blow-up for a system of nonlinear Schrodinger equations was studied in [38] under some conditions on nonlinearities, but it is an open problem for (28).

The asymptotic behavior of solutions to quadratic derivative nonlinear Schrödinger systems has been considered recently in papers [39, 40] under some structural conditions on the nonlinearity. If the structural conditions are not satisfied, the problem is open.
